# Novel mechanism of gene transfection by low-energy shock wave

**DOI:** 10.1038/srep12843

**Published:** 2015-08-05

**Authors:** Chang Hoon Ha, Seok Cheol Lee, Sunghyen Kim, Jihwa Chung, Hasuk Bae, Kihwan Kwon

**Affiliations:** 1 Medical Research Institute, School of Medicine, Ewha Womans University, Seoul, 158-710, Korea; 2 Department of Internal Medicine, Cardiology Division, School of Medicine, Ewha Womans University, Seoul, 158-710, Korea; 3 Department of Asan Institute for Life Sciences, Asan Medical Center, College of Medicine, University of Ulsan, 86 Asanbyeoungwon-gil, Songpa-gu, Seoul, 138-736, Korea; 4 Department of Rehabilitation Medicine, School of Medicine, Ewha Womans University, Seoul, 158-710, Korea

## Abstract

Extracorporeal shock wave (SW) therapy has been studied in the transfection of naked nucleic acids into various cell lines through the process of sonoporation, a process that affects the permeation of cell membranes, which can be an effect of cavitation. In this study, siRNAs were efficiently transfected into primary cultured cells and mouse tumor tissue via SW treatment. Furthermore SW-induced siRNA transfection was not mediated by SW-induced sonoporation, but by microparticles (MPs) secreted from the cells. Interestingly, the transfection effect of the siRNAs was transferable through the secreted MPs from human umbilical vein endothelial cell (HUVEC) culture medium after treatment with SW, into HUVECs in another culture plate without SW treatment. In this study, we suggest for the first time a mechanism of gene transfection induced by low-energy SW through secreted MPs, and show that it is an efficient physical gene transfection method *in vitro* and represents a safe therapeutic strategy for site-specific gene delivery *in vivo*.

Transfection of naked nucleic acids without a viral vector into cultured primary cells and tissues in animal models is increasingly being studied to understand the regulation of gene expression and cognate proteins^1^,^2^. It is also being utilized in various human clinical trials for genetic vaccines, peripheral limb ischemia, and cardiac ischemia^3^,^4^,^5^. As short interfering RNA (siRNA) can inhibit target gene expression in mammalian cells^6^,^7^, it has become increasingly viewed as a potential therapeutic agent for various diseases. However, before it can be used for specific gene silencing in clinical settings, the major challenges of creating site-specific, non-viral transfection agents for use in gene therapies must be solved^8^.

Many physical methods of gene transfection into mammalian primary cells have been developed, including transfection reagents composed of lipids, liposomes or erythrocyte ghosts, as well as electroporation methods^8^,^9^,^10^,^11^,^12^,^13^,^14^. However, these methods have critical limitations for applications in the human body. Therefore, alternative physical gene delivery methods that are effective, safe and site-specific must be investigated.

SW is a sequence of single acoustic waves, similar to ultrasound, conducted through a specific single-sonic generator. It is a single longitudinal pulse with a short duration of less than 1 μs, with an amplitude of high peak pressure up to 100 MPa, and is conveyed by an appropriate generator to a specific target area at an energy density ranging from 0.005–0.32 mJ/mm^2^,^15^,^16^. High-energy extracorporeal shock-wave lithotripsy (ESWL), with peak pressures of 35–100 MPa has been used regularly for the treatment of kidney stones since the 1980s^17^. However, ESWL can trigger the physical phenomenon of cavitation, a major mechanism of tissue damage due to severe structural alterations in cells^18^,^19^,^20^.

Recently, several studies have utilized ultrasound technology, including low energy levels of SW, as a new non-viral physical gene transfer method^1^,^21^,^22^,^23^,^24^,^25^,^26^,^27^. The results suggest that the effect may be due to cavitation or sonoporation, resulting in a transient generation of pores in the cell membrane that allow the direct passage of naked target genes. In this study, we describe a SW-induced gene transfection technique as an effective non-viral physical gene transfer method, both *in vitro* and *in vivo*. Interestingly, HUVECs incubated for 0 to 5 min post-SW treatment were not transfected with siRNAs, whereas HUVECs incubated for 24 h showed high transfection efficiency. We also showed that SW treatment induced the secretion of MPs, and that the gene transfection effect of SW was transferable via the secreted MPs. Based on these findings, we suggest that gene transfection induced by low-energy SW was not by sonoporation, but through secreted MPs.

## Results

### Low-energy level SW showed gene transfection effects on endothelial cells

To develop the SW-induced gene delivery system, an AR2 was used as the single-sonic generator (Dornier MedTech, Wessling, Germany), generating a fixed electromagnetic SW with a diameter of 1 cm and an effective depth of 4 cm^28^. Low energy levels ranging from 0.01–0.04 mJ/mm^2^ had no detrimental effects on HUVECs, however, high lethality effects were observed at energy levels exceeding 0.06 mJ/mm^2^. Therefore, it was determined that SW treatment at 0.04 mJ/mm^2^ was a safe and functional energy level for transfecting siRNAs into primary cultured cells.

**Figure 1:**
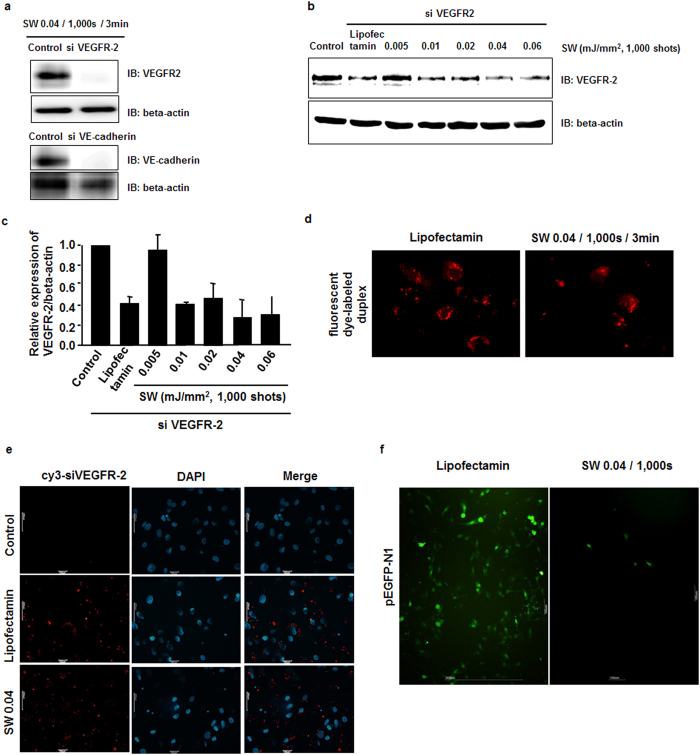
Effects of low-energy level SW on *in vitro* gene transfection into endothelial cells. (**a**–**c**) siRNAs for VEGFR2 and VE-cadherin were added to HUVEC culture medium. Cells were treated with SW at 0.04 mJ/mm^2^ with 1,000 shots for 3 min (**a**) or at the energies indicated with 1,000 shots for 3 min (**b** and **c**). Representative immunoblots and quantitative data are shown (n = 3). *p < 0.05 versus the control group (no SW treatment). Error bars represent standard deviation (SD). (**d**) A fluorescent dye-labeled duplex was added to HUVEC culture medium followed by low-energy level SW treatment (0.04 mJ/mm^2^). (**e** and **f**) HUVECs were transfected with Cy3-labeled VEGFR-2 siRNA (**e**) or a vector encoding full-length enhanced green fluorescence protein (pEGFP-N1, 4.7 kbp) (**f**) by SW treatment (0.04 mJ/mm^2^). Lipofectamine served as the positive control. Cells were fixed, and transfection of siRNAs or the vector was visualized by fluorescence microscopy. Nuclei were stained with DAPI (blue).

The first stage was to confirm the *in vitro* efficacy of SW-induced siRNA transfection into HUVECs (Fig. 1a). siRNAs for VEGFR2 and vascular endothelial cadherin (VE-cadherin) were added to HUVEC culture medium for transfection and treated with SW (0.04 mJ/mm^2^) with 1,000 shots for 3 min, and incubated at 37 °C. Transfection of siRNAs specifically targeting human VEGFR2 and VE-cadherin was dramatically reduced in HUVECs following SW treatment (0.04 mJ/mm^2^), indicating that siRNA transfection in primary cultured cells was induced by SW treatment (Fig. 1a). Next, the transfection efficiency of SW was compared with Lipofectamine. SW in the range of 0.02–0.06 mJ/mm^2^ enabled delivery of VEGFR2 siRNA into HUVECs, compared with Lipofectamine (Fig. 1b,c). The transfection efficiencies of Cy3-labeled VEGFR-2 siRNAs were comparable (Fig. 1b–e).

To further investigate SW-induced plasmid transfection efficiency, HUVECs were transfected with a vector encoding full-length enhanced green fluorescence protein (pEGFP-N1, 4.7 kbp) by SW treatment (0.04 mJ/mm^2^) or Lipofectamine, which served as a positive control (Fig. 1f). The percentage of GFP-positive cells was increased by the high transfection efficiency (from 20–30%) in the Lipofectamine-treated group. However, SW treatment resulted in fewer GFP-positive cells. These results suggest that plasmids may be transfected less efficiently by SW treatment.

### Efficiencies of SW-induced siRNA delivery into various cell lines

To investigate the gene silencing effects of SW-induced siRNA transfection in various cell lines, human smooth muscle cells (HSMCs) and murine colon adenocarcinoma cells (CT26) were transfected with Cy3-labeled GAPDH siRNAs by SW treatment (0.04 mJ/mm^2^) or Lipofectamine.

**Figure 2:**
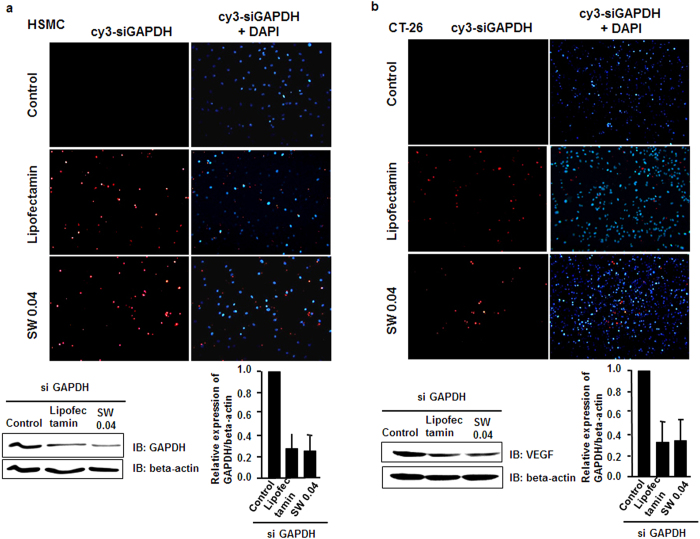
Varying efficiency of SW-induced siRNA delivery in different cell lines. (**a** and **b**) Human smooth muscle cells (HSMCs) or murine colon adenocarcinoma (CT26) cells were transfected with Cy3-labeled GAPDH siRNAs by SW treatment (0.04 mJ/mm^2^) or Lipofectamine (positive control). Cells were fixed, and transfection of siRNAs or the vector was visualized by fluorescence microscopy. Cy3-labeled GAPDH siRNA immunofluorescence staining is indicated in red and DAPI-stained nuclei in blue. Representative immunoblots and quantitative data are shown (n = 3). *p < 0.05 versus the control group without SW treatment. Error bars represent SD.

At 24 h post-SW treatment, HSMCs and CT26 cells showed similar siRNA transfection efficiency to Lipofectamine treatment (Fig. 2a,b). The mean GAPDH protein expression was significantly lower in cells transfected with GAPDH siRNA by SW than in the control groups (p < 0.05). Additional cell lines, including a human prostate cancer cell line (PC-3), immortalized mouse aortic endothelial cells (iMAEC) and monkey kidney fibroblast (COS-7) cells were transfected with Cy3-labeled VEGF, KDR and GAPDH siRNAs by SW treatment (Supplementary Fig. 1). The data suggest that siRNAs were delivered into cells by low-energy SW treatment with suppression of the target gene expression.

### SW-induced gene transfection into mammalian cells may not occur via sonoporation

Recently, several studies focused on the transfection effects of SW by sonoporation, resulting in the transient generation of pores in the cell membrane that allow direct transfection of naked target genes^15^,^19^,^23^,^29^,^30^,^31^.

**Figure 3:**
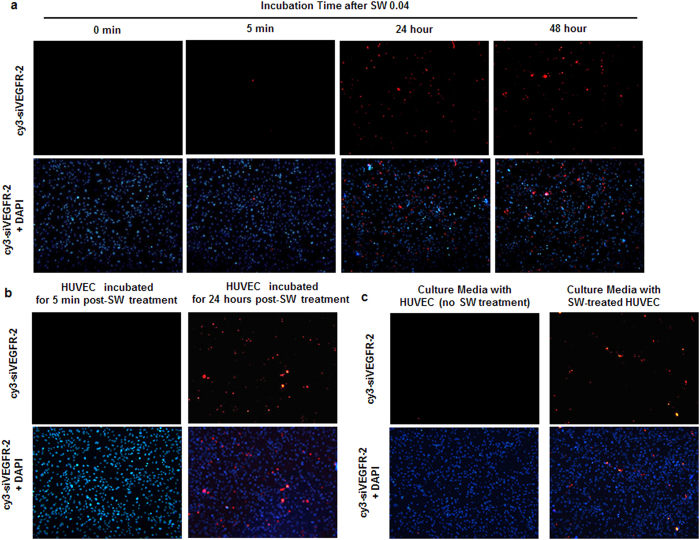
SW-induced transfection without sonoporation. (**a**) Cy3-labeled VEGFR2 siRNAs were added to HUVEC culture medium and treated with SW at 0.045 mJ/mm^2^ prior to incubation for 0 min–48 h. (**b**) SW-stimulated HUVECs were incubated for the indicated times followed by a medium change to remove siRNAs and were then incubated for an additional 24 h. (**c**) Cy3-labeled VEGFR2 siRNAs were added to confluent HUVECs in the absence or presence of SW and incubated for 24 h. After incubation, control and SW-treated HUVEC media were transferred to HUVECs without SW treatment followed by an additional 24-h incubation. Cells were fixed, and transfection of siRNAs or the vector was visualized by fluorescence microscopy. Cy3-labeled VEGFR-2 siRNA immunofluorescence staining is indicated in red and DAPI-stained nuclei in blue.

To confirm whether SW-induced siRNA transfection was caused by sonoporation, Cy3-labeled VEGFR2 siRNAs were added to HUVEC culture medium for transfection and treated with SW (0.04 mJ/mm^2^), followed by incubation for 0 min to 48 h (Fig. 3).

No siRNA was transfected into cells during a 0–5-min incubation following SW treatment. However, siRNAs were delivered successfully into HUVECs following incubation periods of 1, 3, 6, 24 and 48 h post-SW treatment (Fig. 3a and Supplementary Fig. 2), with 24 h being the optimal incubation time. To confirm whether sonoporation by SW treatment was essential for siRNA transfection, the importance of incubation time for siRNA transfection into HUVECs was investigated by changing the SW-treated HUVEC medium. Cy3-labeled VEGFR2 siRNAs were transfected into HUVECs by SW treatment (0.04 mJ/mm^2^). SW-stimulated HUVECs were then incubated for the indicated times and medium changed to remove any remaining siRNAs and incubated for an additional 24 h. Interestingly, HUVECs incubated for 5 min post-SW treatment were not transfected with Cy3-labeled VEGFR2 siRNAs, whereas HUVECs incubated for 24 h showed a very high transfection efficiency of Cy3-labeled VEGFR2 siRNAs (Fig. 3b).

Many studies have suggested that the gene transfection effect of SW treatment is caused by sonoporation after only a few seconds, which allows the direct transfection of naked target genes^19^,^30^,^31^. However, in this study, we showed that SW-induced siRNA transfection required an extended incubation period following SW treatment, indicating that SW-induced gene transfection is not mediated via sonoporation.

To investigate whether SW-induced secretion of the siRNA carriers played an important role in siRNA transfection, confluent HUVECs were added to Cy3-labeled VEGFR2 siRNAs with or without SW treatment, and incubated for 24 h. After 24 h, the medium from SW-treated HUVECs was transferred to non-SW-treated HUVECs on a plate, followed by an additional 24-h incubation (Fig. 3c). Interestingly, while the cells did not undergo SW treatment, the culture medium derived from siRNA and SW-treated cells induced transfection of the naked siRNAs into new cells. Furthermore, to confirm siRNA delivery of secreted MPs, we examined SW-induced transfection efficiency in MP-free medium. HUVECs were treated with SW and Cy3-labeled VEGFR2 siRNAs and incubated for 3 h. The medium was ultracentrifuged at 170,000 × g for 2 h to remove MPs. MP-free medium was then transferred to new HUVECs and incubated for 24 h. The Cy3-labeled VEGFR2 siRNAs were not detected in HUVECs cultured with MP-free media (ultracentrifuged medium). However, the siRNAs were delivered successfully into HUVECs, with secreted MPs observed in the medium (Supplementary Fig. 3). These results demonstrate that the gene transfection effect of SW is transferable via a carrier (for example, MPs or proteins). Our results suggest that SW induces the secretion of MPs, which serve as carriers of siRNAs.

### VEGF-stimulated aorta ring angiogenesis was blocked by SW-induced transfection of VEGF siRNA

**Figure 4:**
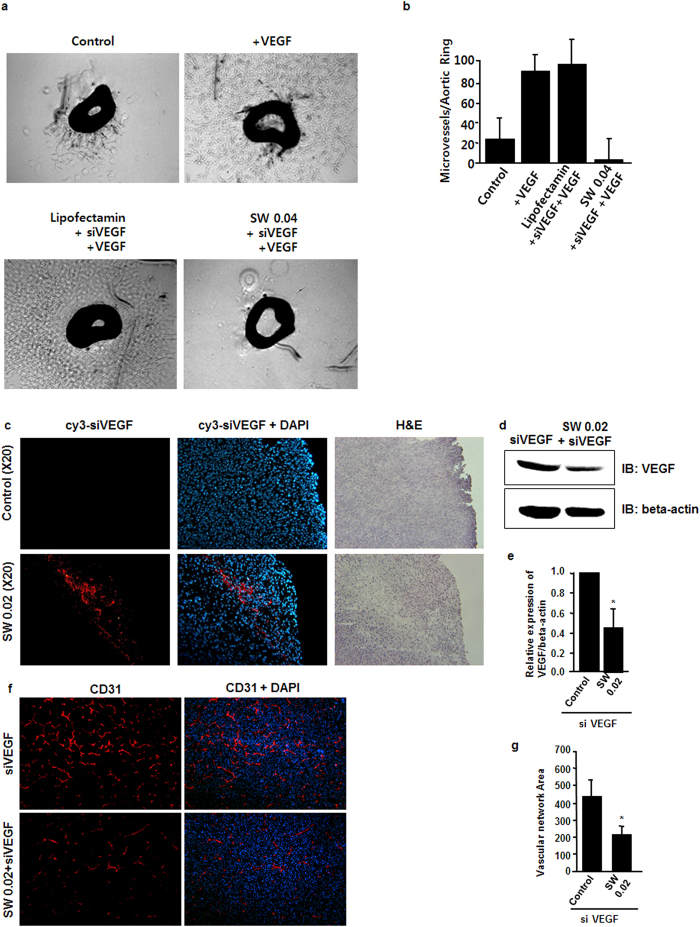
SW-induced transfection of VEGF siRNAs *ex vivo* and *in vivo.* (**a** and **b**) Mouse aorta rings were isolated. VEGFR2 siRNAs were added to the culture medium of aortic rings and treated with SW at 0.045 mJ/mm^2^, incubated for 6bdays, at which point an aorta ring assay for *ex vivo* angiogenesis was performed. VEGF increased the number of microvessels sprouting from the aortic rings. Representative images are shown (n = 4). *p < 0.05 versus the control group (no SW treatment). Error bars represent SD. (**c**) Cy3-labeled VEGF siRNA following SW treatment (0.02 mJ/mm^2^) was transfected into CT26 tumors. (**d** and **e**) Data represent the expression levels of VEGF in CT26 tumors following SW treatment and VEGF siRNA. Representative immunoblots and quantitative data are shown (n = 3). *p < 0.05 versus + VEGF without SW treatment. Error bars represent SD. (**f** and **g**) SW treatment (0.02 mJ/mm^2^) resulted in weak CD31 staining.

To define the efficiency of SW-induced siRNA transfection in intact vessels, an aorta ring assay was conducted for *ex vivo* angiogenesis^32^,^33^. VEGF increased the number of microvessels sprouting from aortic rings isolated from mice, and transfection of VEGF siRNA by SW treatment markedly inhibited the sprouting of VGEF-induced microvessels (Fig. 4a,b). These data suggest that siRNAs was successfully introduced into the cells and functionally worked well in *ex vivo* by SW-induced transfection

### Treatment of CT-26 tumors by *in vivo* SW-induced siRNA transfection

We next investigated whether siRNA delivered into tumor tissues by SW treatment had an inhibitory effect. A Dornier AR2 ESWT (Dornier MedTech) was used to ensure correct orientation of the SW with respect to the horizontal plane. Two weeks after subcutaneous implantation of CT26, tumor-bearing nude mice were treated with Cy3-labeled VEGF siRNA by SW (0.02 mJ/mm^2^). SW treatment at 0.02 mJ/mm^2^ proved to be the optimum condition for successful transfection of siRNAs into the CT26 tumors (Fig. 4c). Many tumor cells were lost at a SW concentration of 0.04 mJ/mm^2^, indicating severe damage to the tissue (data not shown). The control tumor group showed high expression of VEGF, whereas the tumor group treated with SW (0.02 mJ/mm^2^) expressed only small amounts of VEGF (Fig. 4d,e). Furthermore, the control tumor group showed strong CD31 staining (red), which indicated high microvascular density, whereas those treated with SW (0.02 mJ/mm^2^) exhibited weak CD31 staining, indicating decreased microvasculature (Fig. 4f,g).

### Secretion of MPs was induced by low-energy-level SW treatment

**Figure 5:**
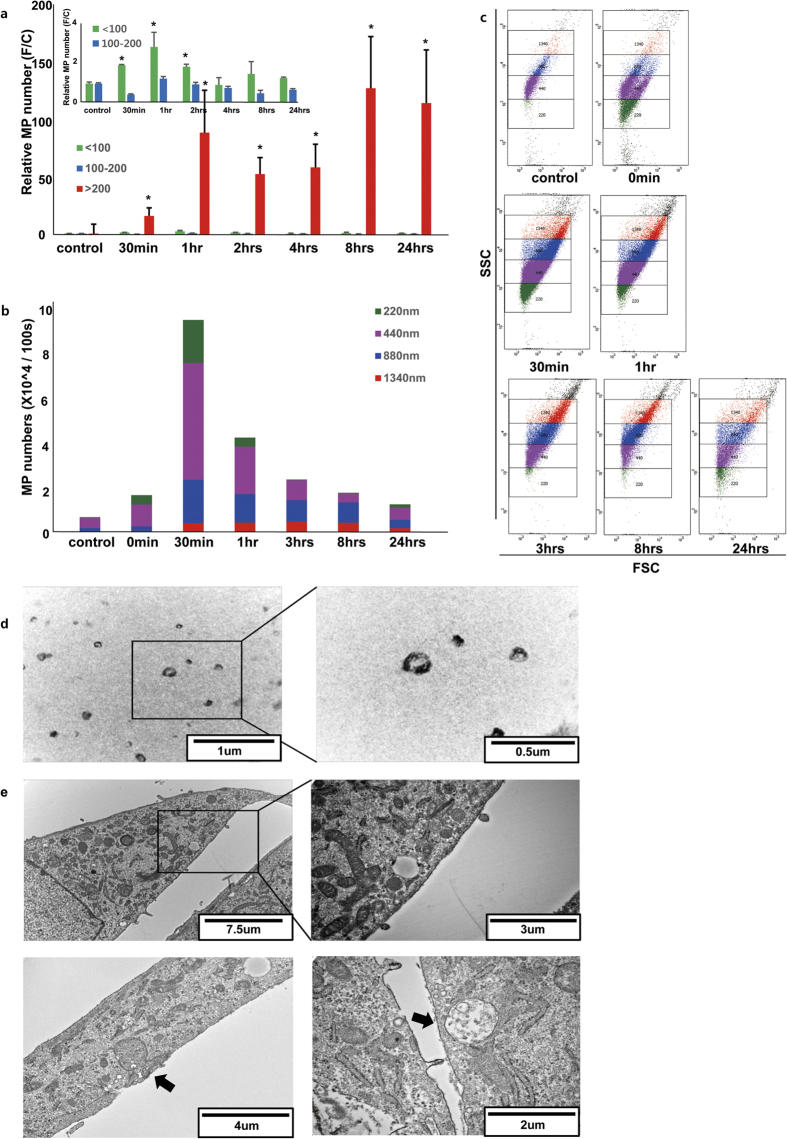
Characterization of MPs isolated from HUVEC culture medium. Size distribution of MPs in culture medium was calculated by flow cytometry and NanoSight particle-tracking analysis (NTA). (**a**) Changes in MP number following SW treatment over time were determined by NTA. Size distribution of MPs; below 100 nm (green), 100–200 nm (blue), over 200 nm (red). All experiments were performed in triplicate. *p < 0.05 versus the control group (no SW treatment). Error bars represent SD. (**b** and **c**) The distribution of MPs in culture medium over time following SW treatment was analyzed by flow cytometry. An MP count was performed for 100 s. Relative sizes were calculated using SPHERO Nano Polystyrene & Nano Fluorescent Size Standard kits. Sizes of 100–300 nm are shown: 220 nm (green), 400–600 nm as 440 nm (purple), 700–900 nm as 880 nm (blue), and 1,000–1,900 nm as 1340 nm (red). (**d**) Representative transmission electron micrographs of MPs obtained from the medium (upper panel) and cell pellets (lower panel). (**e**) The presence of MPs within larger vesicles in the cytoplasm is shown by transmission electron microscopy. Samples were collected 1 h post-SW treatment.

**Figure 6:**
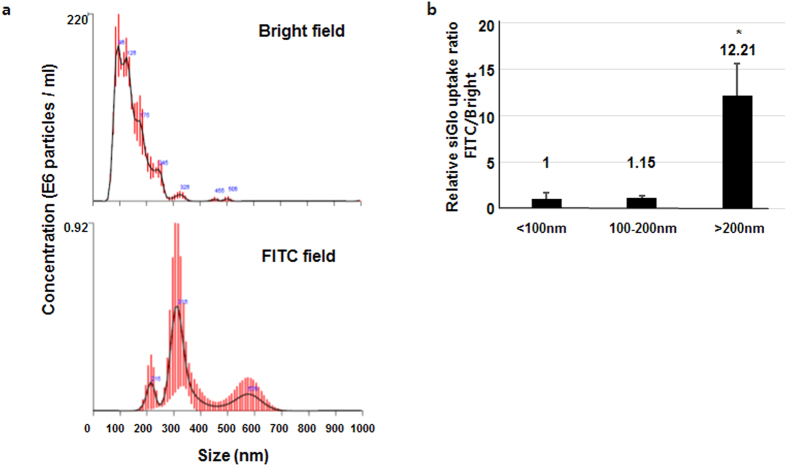
siGlo incorporation and MP secretion following SW treatment. (**a**) Size distribution of MPs in bright field and FITC channel showing siGlo incorporation. Graphs represent the average numbers of MPs of each size. Red error bars indicate ± 1 standard error of the mean. (**b**) Relative siGlo uptake as demonstrated by the ratio of FITC:bright field particles. All experiments were performed in triplicate. *p < 0.05 versus the control group (no SW treatment). Error bars represent SD.

To understand the mechanisms of SW-regulation of the secretion of various MPs from cells, Nanoparticle Tracking Analysis (NTA) was performed using a NanoSight NS300 (Malvern Instruments, Malvern, UK). The results showed an increase in secreted MPs at 30 min to 1 h after SW treatment, followed by a slow decrease. Following SW treatment, a greater number of large-sized particles (>200 nm) relative to smaller particles was revealed (Fig. 5a). FACS analysis was carried out to investigate the large particles using a BD FACS Canto II (Beckman Coulter, Brea, CA, USA); the particles were sized according to standard-sized beads of 220, 440, 880 and 1,340 nm. The beads were run through the flow cytometer using the default settings to collect MP data, and from this, the mean FSC measurement was calculated^34^. Similar to the NTA results, the number of MPs increased significantly at 30 min post-SW treatment and decreased slowly thereafter. These results suggest that SW treatment induces the secretion of various MPs within a short time frame. Furthermore, analysis of the distribution of larger MPs showed a dramatic increase in MPs 220 nm and 440 nm in diameter immediately following SW treatment (Fig. 5b,c and Supplementary Fig. 4). NanoSight and FACS results showed that SW dramatically induced MPs of 200–500 nm diameter within 30 min to 1 h of treatment. Additionally, transmission electron microscopy revealed higher numbers of MPs in the culture medium (Fig. 5d), along with budding of the plasma membrane and multi-vesicular body (MVB) in HUVECs (Fig. 5e). The incorporation of siGlo post-SW treatment was also examined using a NanoSight (Malvern Instruments). The absolute number of MPs with a diameter >200 nm was lower than those with a diameter <200 nm; however, the larger MPs had a siGlo uptake ratio more than 10-fold that of MPs sized ≤100 nm, and of those 100–200 nm (Fig. 6a,b). Taken together with the results shown in Figs 3, 5 and 6, the MPs secreted following SW treatment may interact with naked siRNA and can be transfected into cells not directly treated with SW.

## Discussion

A variety of methods have been used for nucleic acid transfection into primary cells and tissues. Viral vectors have proven to be the most efficient and effective gene delivery method. However, viral vectors have important limitations in terms of safety, such as toxicity, immunogenicity, and the presence of oncogenes from insertional mutagenesis^35^,^36^. Viral vectors also have innate tropisms to specific cell types or cell-selective promoters, which may limit their effectiveness in other cell types^37^.

Non-viral methods can overcome most of the limitations associated with viral gene delivery methods. However, non-viral methods exhibit lower delivery efficacies. Of the non-viral approaches, chemical vectors—such as cationic lipids, cationic polymers, and cell-penetrating peptides—overcome some of the safety concerns due to the infectivity of viral vehicles; however, effective doses of chemical vectors can be toxic, especially to sensitive cell populations because large doses are required to overcome the poor efficiency^38^. On the other hand, physical methods—such as microinjection, ballistic gene delivery, electroporation, sonoporation, and laser irradiation—have been shown to be effective for transfecting primary cells, progenitor cells, and stem cells through *in vitro*, *ex vivo*, and *in vivo* approaches^39^. However, depending on the physical delivery method used, the cell may sustain heavy trauma and initiate apoptotic mechanisms. Hence, low cell viability is a major problem that limits physical gene delivery into cells and tissues^40^.

Here we show that a low-energy-level SW, as a non-viral physical gene transfection method, can accomplish effective and safe gene transfection. Our strategy of siRNA transfection by SW treatment resulted in sufficient delivery and efficiency in primary cells such as HUVECs, HSMCs, tumor cells and even in tumor tissues *in vivo*. Transfection of siRNAs for VEGF using SW resulted in significant suppression of angiogenesis and VEGF expression in tumor tissues. Unlike other physical gene transfection methods, such as electroporation and sonoporation, low-energy-level SW did not trigger apoptosis mechanisms, as described in our previous study^28^. Our experiments were conducted to assess the degree of strength required not to fall such that apoptosis did not progress^28^. Gene transfection using SW has another advantage, namely, high site-specific delivery. The SW probe is usually <1 cm in diameter, and can deliver genes into small tumors or specific sites of a solid organ in a clinical setting.

In the mid 1990s, several papers showed that ultrasound, including low-energy SW, could facilitate the transport of membrane impermeable compounds into living cells. This included reports that ultrasound induced the uptake of low-molecular-weight drugs, nucleic acid-based drugs (pDNA, siRNA, mRNA), peptides and proteins^40^,^41^,^42^,^43^. In general, the uptake of these drugs or nucleic acids was attributed to ultrasound-mediated transient permeabilization of the cell membrane. The first studies on ultrasound-induced cell permeabilization used the term “sonoporation” to describe temporal cell membrane openings that arose after exposure to ultrasound^44^,^45^,^46^.

However, we suggest that the mechanism underlying SW-mediated gene transfection was not sonoporation. Cy3-labeled VEGFR2 siRNA and SW-treated HUVECs were incubated for the indicated times, followed by a medium change to remove the siRNAs. HUVECs incubated for 0–5 min post-SW treatment were not transfected with siRNAs, whereas those incubated for 24 h post-SW treatment showed a high transfection efficiency. If the mechanism of gene transfection were sonoporation, peak transfection would have occurred immediately after SW treatment. In a previous study, the pores induced by sonoporation resealed in <3 min^47^.

Furthermore, in this study the gene transfection effect of SW was transferable via the culture medium without SW treatment. The culture medium from siRNA- and SW-treated cells induced transfection of the naked siRNAs into new cells with no need for SW treatment. Therefore, we speculated the possibility of SW-induced secretion of specific siRNA carriers from cells. SW treatment induced the secretion of MPs of various sizes in a time-dependent manner. The larger MPs (>200 nm) were capable of taking up a greater quantity of siRNAs than the smaller MPs. Taken together, these results indicate that SW treatment induces the release of MPs as siRNA carriers from cells, and that these secreted MPs possibly interact with naked siRNA; this may be via a transferable gene transfection effect of SW. Although, additional studies will be required to clarify the mechanism underlying the secretion of MPs following SW therapy as well as the interaction between MPs and nucleic acids, these results suggest that low-energy SW could be developed as a remote or systemic gene delivery system.

Recently, lung-specific delivery of siRNA using a cationic fullerene, tetra(piperazino) fullerene epoxide (TPFE), was published. This delivery agent agglutinated with siRNA and plasma proteins in the bloodstream to form >6-μm particles, which accumulated in narrow lung capillaries^48^. Moreover, methods using modified exosomes that specifically target the brain by expressing a brain-targeting peptide (rabies virus glycoprotein peptide : RVG) were also published^49^. These methods likely benefit gene delivery to a specific target. However, they are not easy to use in a variety of targets or organs.

In summary, we showed that low-energy SW was an effective approach for non-viral physical gene transfection in various primary cells, as well as *in vivo* tumor tissues. We suggest that SW-mediated gene transfection is mediated not by sonoporation or cavitation but through the secretion of MPs as carriers. Furthermore, we found that the gene transfection effect of SW was transferable, probably through the secreted MPs functioning as siRNA carriers. Therefore, we suggest that low-energy SW represents a safe and effective non-viral gene transfection technique for site-specific- or systemic gene delivery in various clinical settings.

## Methods

### Cell culture and SW treatment

HUVECs were isolated from fresh human umbilical veins and HSMCs were purchased from Gibco (Inchinnan, Scotland, UK). HUVECs and HSMCs were grown in Medium 200 with 5% fetal bovine serum (FBS) and low-serum growth supplement (LSGS; Cascade Biologics Inc., Winchester, MA, USA)^50^. FBS containing MPs were ultracentrifuged at 170,000 × g for 2 h to remove all MPs. Immortalized murine aortic endothelial cells (iMAECs) were kindly provided by Dr. Hanjoong Jo (Emory University, Atlanta, Georgia, USA) and grown in Dulbecco’s Modified Eagle Medium (DMEM) supplemented with 10% FBS, 1% penicillin-streptomycin, 50 μg/mL EC growth supplement (Sigma Aldrich, St. Louis, MO, USA), and 1X EMEM with non-essential amino acids.

For *in vitro* SW treatment, confluent cells were cultured in a 12-well plate and treated with SW using a Dornier AR2 ESWT (Dornier MedTech, Germany)^28^. Briefly, cultured cells were directly subjected to SW treatment by perpendicularly immersing a sterile SW probe into the well plate such that it contacted the surface of the medium. The distance between the SW probe and cell layer on the dish bottom was 1.2 cm. Cultured cells were exposed to 1,000 shots of SW at the indicated energy levels for 3 min and incubated in a CO2 incubator at 37 °C. Control cells were prepared in the same manner in the absence of SW treatment. Cells were then harvested and subjected to Western blot analysis. Cells were fixed, and transfection of siRNAs or vector was visualized by fluorescence microscopy. Cy3-labeled VEGFR-2 siRNA immunofluorescence staining is indicated in red and DAPI-stained nuclei in blue.

To assess whether sonoporation by SW treatment is essential for siRNA transfection, SW-stimulated HUVECs were incubated for the indicated times, followed by a medium change to remove the siRNAs and then incubated for another 24 h.

Media transfer methods were performed as follows. Cy3-labeled VEGFR2 siRNAs were added to confluent HUVECs, followed by SW treatment and incubated for 24 h. After incubation, control and SW-treated HUVEC media were transferred to HUVECs without SW treatment and incubated for 24 h. All experimental protocols were approved by Ewha Woman’s University.

### Reagents and antibodies

VEGFs were purchased from R&D System, Inc. (Minneapolis, MN, USA). Anti- VEGF, VEGFR-2 and VE-Cadherin (C-19) antibodies were obtained from Cell Signaling Technologies (Beverly, MA, USA). Anti-β-actin and GAPDH antibodies were purchased from Santa Cruz Biotechnology (Dallas, TX, USA).

### SDS-PAGE and Western blot analysis

Cells were harvested with lysis buffer (0.5% Triton X-100, 0.5% Nonidet P-40, 10 mM Tris, pH 7.5, 2.5 mM KCl, 150 mM NaCl, 30 mM glycerophosphate, 50 mM NaF, and 1 mM Na_3_VO_4_) containing a 0.1% protease inhibitor mixture (Sigma) and clarified by centrifugation. The protein concentration in the lysates was determined using the Bradford method (Bio-Rad, Hercules, CA, USA). Protein complexes were separated by sodium dodecyl sulfate polyacrylamide gel electrophoresis (SDS-PAGE) and transferred to nitrocellulose membranes. Membranes were then incubated with the appropriate primary antibodies. After washing and incubating with secondary antibodies, immunoreactive proteins were visualized using an enhanced chemiluminescence (ECL) detection system (Amersham Biosciences, Amersham, UK). Where indicated, the membranes were stripped and reprobed with another antibody. Densitometric analyses of immunoblots were performed using ImageJ software (the National Institutes of Health). Results were normalized by arbitrarily setting the densitometry of the control cells to ‘1.0’.

### Transfection of small interfering RNA (siRNA)

The siRNA duplex targeting VEGFR2, vascular endothelial-cadherin (VE-cadherin), GAPDH, and the scrambled siRNA control (a non-targeting siRNA pool) were purchased from Integrated DNA Technologies (IDT; Coralville, IA, USA). The sequences of siRNA against human VEGFR2 were as follows: sense 5′- ACAAUGACUAUAAGACAUGCUAUGG and antisense 5′- CCAUAGCAUGUCUUAUAGUCAUUGUUC. The sequences of siRNA against human VE-cadherin were as follows: 5′- GCAAUAGACAAGGACAUAACACCAC and antisense 5′- GUGGUGUUAUGUCCUUGUCUAUUGCGG. For the SW-induced transfection of siRNA, cells were seeded into 6-well plates for 24 h at ~80% confluence, and siRNA was transfected by SW at the indicated energy levels with 1,000 shots for 3 min, followed by incubation at 37 °C. Cells were harvested 24 h post-SW treatment. Lipofectamine 2000 (Invitrogen, Waltham, MA, USA) was used as the positive control for siRNA transfection as described previously^51^.

### Aorta ring assay for *ex vivo* angiogenesis

Mouse aortic ring angiogenesis assays were performed as described previously^52^,^53^. Briefly, thoracic aortas from mice (C57BL/6J, 8 weeks, male) were dissected and the periaortic fibroadipose tissue removed under a stereo dissection microscope using fine-tipped forceps and microdissection scissors. Aortic rings (1 mm in length) were embedded in growth factor-reduced Matrigel supplemented with 20 U/mL heparin. Aortic rings were treated by the addition of siRNA or VEGF into the medium in the absence or presence of SW. Cultures were incubated at 37 °C in 5% CO_2_ for 8 days for optimal microvessel sprouting. Aortic ring images were obtained using an Olympus BX41 microscope (Olympus, Center Valley, PA, USA).

### CT26 xenograft tumor mouse model and exposure to SW

This animal study was performed in accordance with the Guidelines for Animal Experiments of the Committee on Ethics in Animal Experiments of Ewha Woman’s University. A total of 4.0 × 10^6^ CT26 cells in 0.3 mL phosphate-buffered saline (PBS) were inoculated subcutaneously using a 24-gauge needle into the lower flanks of 8-week-old nude mice (Central Lab Animal Inc., Seoul, Korea). After 2 weeks when the tumors had reached an average volume of 1 cm^3^, CT26 tumor-bearing nude mice were directly injected with Cy3-labeled VEGF siRNA and treated with SW (0.02 mJ/mm^2^, 1,000 shots). A diluted Cy3-labeled VEGF siRNA solution in 20 μL sterile PBS was directly injected into each tumor. Cy3-labeled VEGF siRNA without SW treatment was injected as a control. The relative fluorescence (red, Cy3-labeled siRNA; blue, nucleus) of sections was visualized using a fluorescence microscope system (Olympus).

### Assessment of intratumor microvessel density

Immunofluorescence staining for CD31 and counting of CD31-positive microvessels were carried out as described previously^54^,^55^,^56^.

### Preparation of SW-treated samples and FACS analysis

For *in vitro* SW studies, confluent HUVECs were cultured in 35 mm dishes and treated with SW, as described above. Control cells were prepared in the same manner but in the absence of SW treatment. HUVECs and culture medium were collected following SW exposure for RT-PCR, Western blot- and FACS analysis. For FACS analysis, harvested samples were centrifuged at 180 × g for 10 min to remove cell debris, and the supernatant was transferred to a new tube and centrifuged again at 1,500 × g for 10 min to remove larger-sized particles. Supernatants were moved to 5-mL polystyrene round-bottomed tubes for FACS analysis. The size and number of MPs were measured using a BD FACSCanto II (Beckman Coulter). MP sizes were determined using SPHERO™ Flow Cytometry Nano Polystyrene and Nano Fluorescent Size Standard Kits (Spherotech, Lake Forest, IL, USA)^57^,^58^,^59^. MPs were counted both manually for 100 s and automatically to reduce errors.

### NanoSight analysis

Microvesicles were analyzed using the NanoSight NS300 system (Malvern Instruments), which allows tracking of the Brownian motion of nanoparticles in a liquid suspension on a particle-by-particle basis. Nanoparticle Tracking Analysis (NTA) 3.0 software was used to analyze the concentrations and sizes of microvesicles^60^. Sample preparation was the same as for FACS analysis, and samples were diluted 1:10.

### Electron microscopy

Cells were perfused with 2% glutaraldehyde and 2% paraformaldehyde in 0.1 M phosphate buffer. The prepared cell blocks were post-fixed in 2% osmium tetroxide, dehydrated, and embedded in epoxy resin. Appropriate areas of interest were selected from sections approximately 1 μm thick and stained with toluidine blue. Approximately 60 × 60-nm ultra-thin sections were cut using an ultramicrotome (Reichert-Jung/Leica Microsystems, Wetzlar, Germany) with a diamond knife. Thin sections were stained with 1–2% aqueous uranyl acetate, followed by 1% lead citrate. Stained sections were observed and photographed with an H-7650 transmission electron microscope (Hitachi, Tokyo, Japan) at an accelerating voltage of 80 kV.

### Statistical analysis

All data are expressed as the means ± standard error of the mean (SEM) of at least three independent experiments for a given sample. The statistical significance of the differences between two groups was assessed by Mann-Whitney U-test. A P value ≤0.05 was considered to indicate statistical significance, and data are reported as means ± the standard error of the mean.

## Additional Information

**How to cite this article**: Hoon Ha, C. *et al.* Novel mechanism of gene transfection by low-energy shock wave. *Sci. Rep.* **5**, 12843; doi: 10.1038/srep12843 (2015).

## Supplementary Material

Supplementary Information
