# Supplementary material for: Novel mechanism of gene transfection by low-energy shock wave
Source: Sci Rep. 2015 Aug 5;5:12843. doi: 10.1038/srep12843 (PMC4525295; doi:10.1038/srep12843)
Supplement: Supplementary Information [file srep12843-s1.pdf]

Novel mechanism of gene transfection by low-energy shock wave

Chang Hoon Ha[3,*], Seok Cheol Lee[1,*], Sunghyen Kim[1], Jihwa Chung[1], Hasuk Bae[4] and Kihwan Kwon[1,2]

[1]Medical Research Institute, School of Medicine, Ewha Womans University, Seoul,158-710, Korea.

[2]Department of Internal Medicine, Cardiology Division, School of Medicine, Ewha Womans University, Seoul, 158-710, Korea

[3]Department of Asan Institute for Life Sciences, Asan Medical Center, College of Medicine, University of Ulsan, 86 Asanbyeoungwon-gil, Songpa-gu, Seoul, 138-736, Korea

[4]Deptartment of Rehabilitation Medicine, School of Medicine, Ewha Womans University, Seoul, 158-710, Korea

*C.H.H. and S.C.L. contributed equally for this study

Address correspondence to: Kihwan Kwon, Department of Internal Medicine, Cardiology Division, School of Medicine, Ewha Womans University, Seoul, 158-710, Korea. Phone: 82-2-2650-2023. Fax: 82-2-2650-2567. E-mail: kankadin@ewha.ac.kr

Supplementary Information

Supplementary Figure 1. Differential efficiency of SW-induced siRNA delivery in various cell lines

(a) PC3, (b) iMAEC and (c) COS-7 cells were transfected with Cy3-labeled VEGF, KDR and GAPDH siRNAs by SW treatment or via Lipofectamine transfection (positive control). Cells were fixed, and transfection of siRNAs was visualized by fluorescence microscopy. Cy3-labeled GAPDH siRNA immunofluorescence staining is indicated in red and DAPI-stained nuclei in blue.

Supplementary Figure 2. SW-induced transfection without sonoporation

Cy3-labeled VEGFR2 siRNAs were added to HUVEC culture medium and treated with SW (0.04 mJ/mm^2) prior to incubation for 1, 3 and 6 h.

Supplementary Figure 3. SW-induced transfection using MP-free medium

HUVECs were treated with SW and Cy3-labeled VEGFR2 siRNAs and incubated for 3 h. The medium was ultracentrifuged at 170,000 x g for 2 h to remove all MPs. MP-free medium was transferred to new HUVECs and incubated for an additional 24 h.

Supplementary Figure 4. Numbers of MPs in control and SW-induced culture media.

The number of MPs in culture medium post-SW treatment over time was measured by flow cytometry. MPs were counted for 100 s. All experiments were performed in triplicate. *$p < 0.05$ versus the control group (no SW treatment). n.s. non-significant. Error bars represent SD.

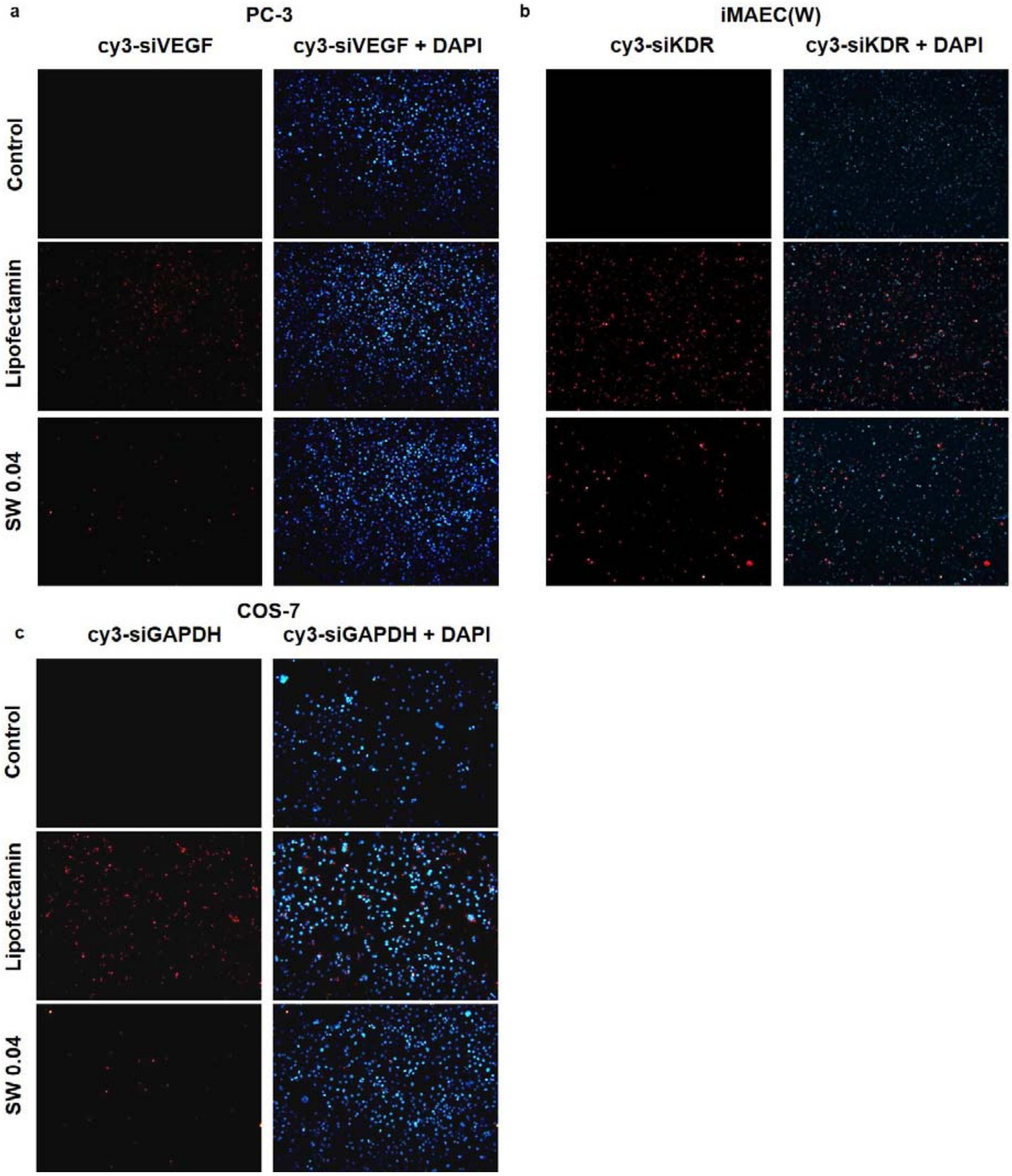

Supplementary Figure 1

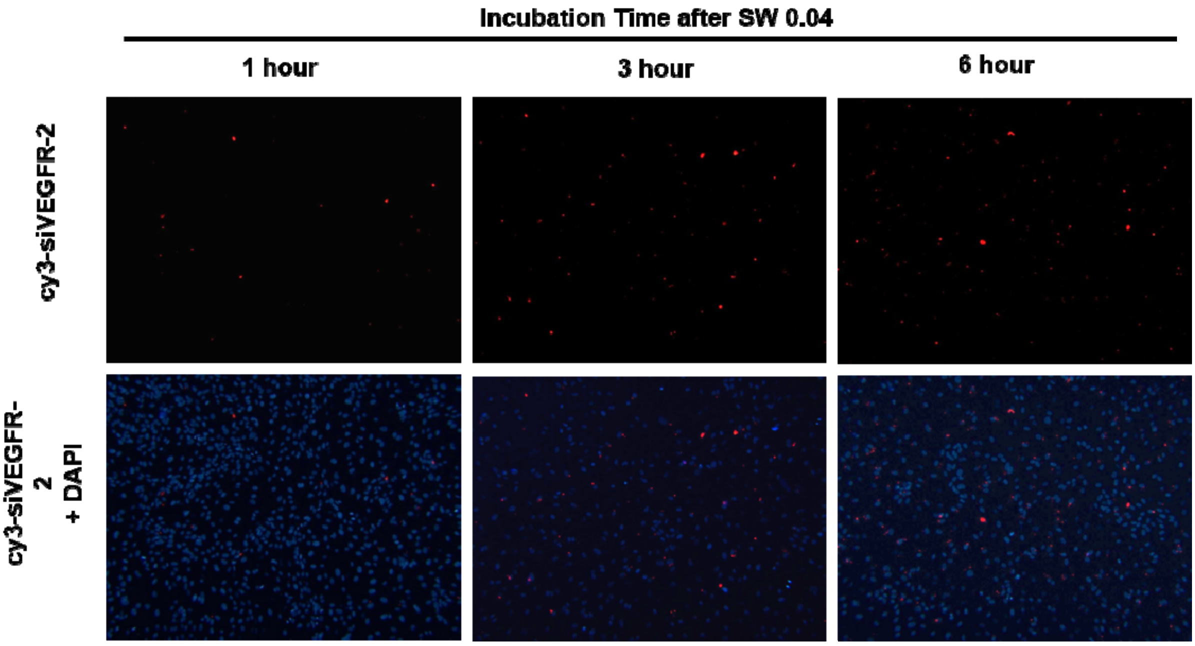

Supplementary Figure 2

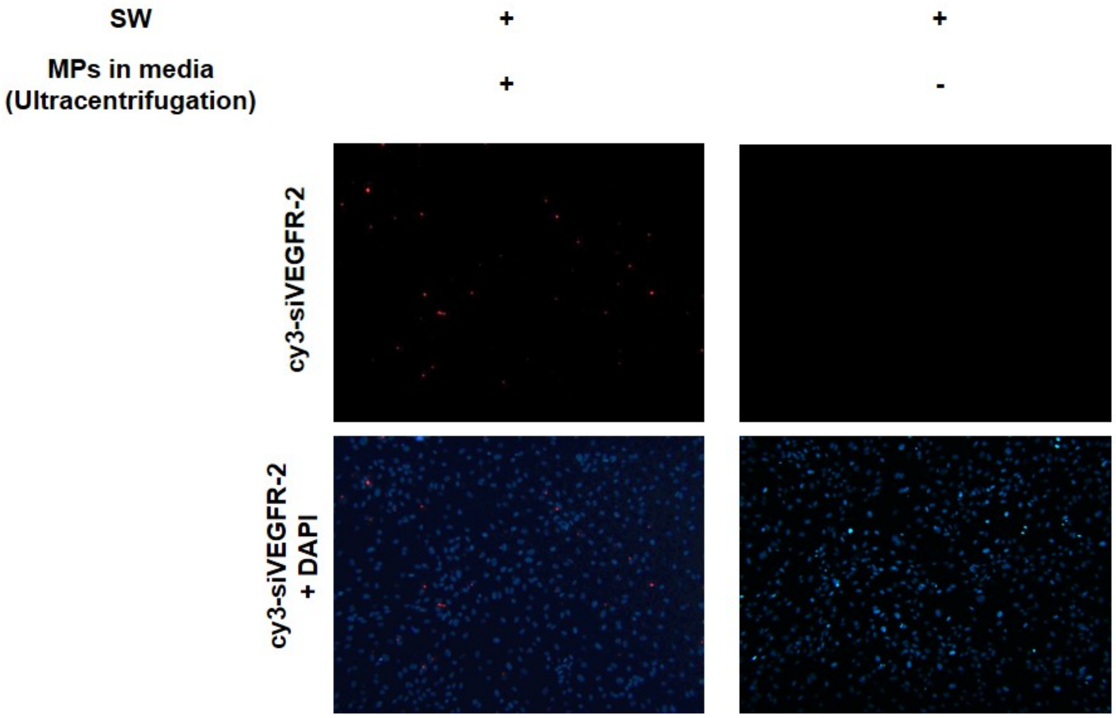

Supplementary Figure 3

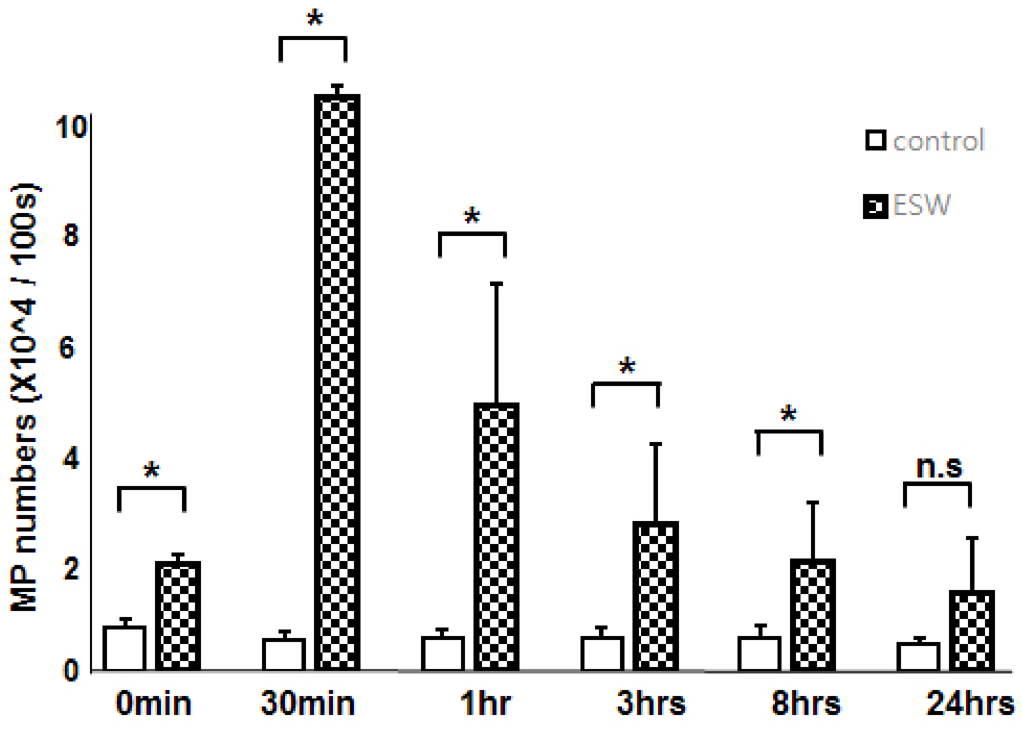

Supplementary Figure 4
